# Cognitive functioning in young adults after mild COVID-19: A case-control study from Iran

**DOI:** 10.1016/j.ibneur.2025.06.003

**Published:** 2025-06-05

**Authors:** Farzad Akbarzadeh, Farhad Faridhosseini, Mahboubeh eslamzadeh, Mojtaba ghalandarzadeh, Saeedeh Hajebikhaniki, Alireza Ebrahimi

**Affiliations:** a Psychiatry and Behavioral Sciences Research Center, Mashhad University of Medical Sciences, Mashhad, Iran; b Department of Biostatistics, Faculty of Health, Mashhad University of Medical Sciences, Mashhad, Iran

**Keywords:** Cognitive impairment, Processing speed, Coronavirus, Attention

## Abstract

**Introduction:**

Since December 2019, the novel coronavirus (COVID-19) has caused widespread infection across global populations, characterized by its high transmissibility. Despite extensive research on the acute effects of COVID-19, the long-term psychological and neurological sequela remain inadequately explored. This study aimed to investigate cognitive function after COVID-19 infection compared to a control group of non-infected subjects.

**Methods:**

This case-control study included 40 individuals who had recovered from COVID-19, referred to Imam Reza Referral and Educational Hospital in Mashhad, Iran, and 40 matched controls who had not experienced COVID-19 symptoms. All participants underwent an initial screening by a psychiatric assistant to exclude significant medical and psychiatric conditions and any history of drug use. A demographic checklist was administered, followed by cognitive assessments using the Stroop Test, Digit Span Test, and Wisconsin Card Sorting Test (WCST). Data were analysed using SPSS Version 20.

**Results:**

No significant differences were observed between the two groups regarding age, sex, education level, marital status, or employment status (p > 0.05). However, COVID-19-infected individuals exhibited significantly longer completion times for the congruent Stroop test and increased reaction times compared to healthy controls (p < 0.05). Additionally, the duration for completing the Wisconsin Card Sorting Test was significantly longer in the infected group compared to the non-infected group (p < 0.001). Although the longest digit span and scores on the Digit Span Test were lower in the infected group, these differences did not reach statistical significance (p > 0.05). Furthermore, reaction times in the Continuous Performance Test (CPT) for the first, second, and third sets of 50 stimuli were significantly greater in the COVID-19 group (p < 0.001).

**Conclusion:**

This study underscores that cognitive performance post-COVID-19 is adversely affected, particularly in terms of processing speed and sustained attention, when compared to healthy individuals. Further research is warranted to elucidate the underlying mechanisms and to explore potential interventions for cognitive rehabilitation in this population.

## 1 Introduction

The emergence of severe acute respiratory syndrome coronavirus 2 (SARS-CoV-2) in late 2019 triggered the coronavirus disease 2019 (COVID-19) pandemic. As of January 25, 2023, the World Health Organization (WHO) had reported over 665 million confirmed cases and, tragically, more than 6.7 million deaths attributed to COVID-19 globally. The disease manifests a wide spectrum of clinical symptoms, ranging from complete absence of symptoms (asymptomatic) to fatal outcomes. While fever and respiratory symptoms, including cough and shortness of breath, are hallmark features of the disease (Baj et al., 2020), a growing body of evidence suggests significant multi-organ involvement beyond the respiratory system emerging evidence suggests significant multi-organ involvement beyond the respiratory system (Thakur et al., 2021).

Initial research primarily focused on respiratory ailments, particularly acute respiratory distress syndrome (ARDS), which can lead to mortality in severe cases. However, as confirmed cases continued to rise, clinical studies began to reveal a broader pathological spectrum. SARS-CoV-2 can affect various organ systems, including cardiovascular, gastrointestinal, hematological, pulmonary, renal, dermatological, and neurological systems (Nakayama et al., 2023). Recent investigations indicate a concerning prevalence of neurological manifestations among COVID-19 patients; over 33 % of infected individuals exhibit acute neurological symptoms, while 34 % show abnormalities on brain imaging, such as white matter hyperintensities, hypodensities, microhemorrhages, and infarcts (Helms et al., 2020, Kumar et al., 2021).

Post-acute sequelae of COVID-19 (PASC), or long COVID, present significant healthcare challenges due to their potential for widespread morbidity (Yang and Tebbutt, 2023). Recent data suggest that approximately 80 % of infected individuals experience at least one persistent symptom (Alkodaymi et al., 2022) and between 10 % and 35 % report a broader spectrum of symptoms months after an initially mild infection (van Kessel et al., 2022). These long-term effects—commonly including fatigue, shortness of breath, and cognitive dysfunction—can significantly impair daily activities and quality of life (Ariza et al.). Cognitive deficits have been documented across various domains, including attention, executive function, working memory, language expression, and learning, particularly among older adults (Cipolli and Alonso, 2023, Velichkovsky et al., 2023). Although many individuals demonstrate spontaneous cognitive recovery post-infection, a concerning subset—especially older adults and those with comorbidities—experience enduring cognitive impairment (Peskar et al., 2023, Zhao et al., 2023). Further research is essential to elucidate the mechanisms underlying these neurological sequelae and to develop effective interventions for those affected.

Throughout the COVID-19 pandemic, the focus on general medical complications has overshadowed the limited research into the direct psychological and neurological consequences of SARS-CoV-2 infection. As such impairments can significantly affect functional independence, work performance, and overall quality of life (Ariza et al.), the early and accurate diagnosis of these symptoms is critical for designing effective interventions aimed at mitigating the long-term effects on cognitive and brain health. This study aims to compare cognitive function in individuals who have experienced COVID-19 infection with those who have not been infected.

## 2 Methods

### 2.1 Ethics

The study protocol was approved by the Ethics Committee of Mashhad University of Medical Sciences, Mashhad, Iran, under ethical number IR.MUMS.MEDICAL.REC.1401.308. All study procedures adhered to the principles of medical ethics as outlined by the Ministry of Health and Medicine, the Declaration of Helsinki, and the Medical Ethics Committee of Mashhad University of Medical Sciences. Patient information was recorded using unique codes to ensure confidentiality. All participants provided informed consent and were assured that they could withdraw from the study at any time.

### 2.2 Study population

This cross-sectional descriptive study was conducted at Imam Reza Teaching Hospital, Mashhad University of Medical Sciences, Mashhad, Iran, during the first six months of 2022. The study included forty individuals who tested positive for COVID-19 via PCR and subsequently tested negative after a minimum of two weeks post-infection. A control group was composed of individuals who had not experienced COVID-19 symptoms and had negative PCR tests.

Inclusion criteria for the study were as follows: participants aged 18–50, literate enough to understand and complete the questionnaire and tests, a minimum of four months post-infection for the case group, and a diagnosis of mild COVID-19 based on WHO guidelines. Participants were required to provide consent to participate in the study. Exclusion criteria included significant neurological, internal, or psychiatric disorders, use of medications affecting cognitive function, and substance abuse.

### 2.3 Study procedure

In this cross-sectional descriptive case-control study, forty individuals who had recovered from COVID-19 and were referred to Imam Reza Teaching Hospital during the first six months of 2022 were included through convenience sampling. The control group consisted of individuals who met the inclusion criteria, had not experienced any symptoms of the disease, and had negative PCR tests for COVID-19.

Following the acquisition of informed consent, all participants were evaluated by a psychiatry resident for the presence of significant internal and psychiatric disorders, as well as for the use of medications or narcotics that could impair cognitive function. Upon confirmation of eligibility, participants completed a demographic questionnaire that collected information on age, gender, marital status, education, occupation, number of COVID-19 infections, and number of vaccine doses received. Subsequently, cognitive assessments were conducted using the Stroop test, Digit Span test, Continuous Performance Test (CPT), and Wisconsin Card Sorting Test. The data obtained from study participants were analyzed using SPSS version 22 software.

### 2.4 Instruments

#### 2.4.1 Wisconsin

The Wisconsin Card Sorting Test is designed to assess various cognitive functions, including attention, abstract thinking, strategic flexibility, perseverance, and working memory. The test consists of 64 cards featuring images that vary by color (red, yellow, blue, or green), shape (cross, circle, triangle, or star), and number (from one to four). These variables create a total of 64 distinct combinations. Scoring can be conducted in several ways, with the most common metrics being the number of categories completed and the number of errors made. The categories represent the number of card sets successfully completed during the test, which can range from six to zero, indicating the participant's progress and ability to identify a series of six rules. A perseverative error occurs when a participant continues to apply a previously correct rule after ten correct responses following a rule change, indicating a lack of cognitive flexibility.

The validity of the Wisconsin test for measuring cognitive deficits associated with frontal lobe damage, such as that seen in schizophrenia, is reported to be above 86 %. The test's reliability is estimated at 83 % based on inter-rater agreement (Nyhus and Barceló, 2009), and in the Iranian population, the reliability has been reported as 85 % using the retest method (Lezak, 2004).

#### 2.4.2 Stroop

The Stroop Test is based on the Stroop effect, which refers to the delay in response to congruent versus incongruent stimuli. This test evaluates selective attention, information processing speed, and overall executive function.

In the first stage of the test, participants are instructed to match a color circle displayed on a computer monitor (in one of four colors: red, blue, yellow, or green) with corresponding keys labeled with those colors on the keyboard. This stage serves as a training phase, and performance does not impact the overall results. In the second stage, participants are presented with 48 congruent and 48 incongruent color words. Congruent words are those where the color matches the word's meaning (e.g., the word "blue" displayed in blue), while incongruent words have mismatched colors and meanings (e.g., the word "blue" displayed in red). A total of 96 color words are displayed randomly on the screen, and participants must identify the color of the text without considering the word's meaning. Each stimulus is presented for 2 s, with an 800-millisecond interval between stimuli. Researchers believe this color-word task measures mental flexibility, interference, and response inhibition. The interference score is calculated by subtracting the number of correct responses for incongruent stimuli from the number of correct responses for congruent stimuli. Measured variables include errors, omissions, correct responses, interference time, and interference score across both congruent and incongruent conditions (Chen et al., 2001, Davidson et al., 2003, Moering et al., 2004).

The reliability of the Stroop test has been reported as 0.01 and 0.90 for the two stages, respectively (Stroop, 1992) In the Iranian population, retest reliability for both stages has been reported as 0.60 and 0.97, respectively.

#### 2.4.3 Digit span

The Digit Span test assesses short-term verbal memory and working memory. It is a subscale of the Revised Wechsler Adult Intelligence Scale (WAIS-R), for which Wechsler (1981) reported a validity coefficient of 0.97 for general and verbal intelligence and 0.93 for practical intelligence. In Iran, the reliability of this subscale was found to be 0.69 to 0.74 using the Cronbach alpha method (Abedi, 1994).

In the forward digit span test, participants repeat a sequence of digits presented by the examiner in the same order. After two successful trials, the number of digits is increased sequentially (one, two, three digits, etc.). The score corresponds to the length of the last successfully repeated sequence. For example, if a participant successfully repeats a sequence of five digits, they receive a score of five. In the backward digit span test, participants repeat the digits in reverse order, with the number of digits decreased after two unsuccessful attempts. Higher scores in both the forward and backward tests indicate better working memory capacity.

#### 2.4.4 Continuous performance test

The Continuous Performance Test is a widely used laboratory tool for measuring attention and is particularly effective in evaluating hyperactivity and attention deficit disorders. This test assesses sustained attention, which refers to an individual's ability to focus on a specific stimulus over a designated period. Various versions of the test have been developed for therapeutic and research purposes. The Persian version, administered via computer, consists of 150 Persian numbers as stimuli, with 30 (20 %) designated as target stimuli. The interval between stimulus presentations is 500 ms, and each stimulus is displayed for 150 ms. The computer records errors, distinguishing between omission errors (related to inattention) and commission errors (related to impulsivity). A higher test score indicates better sustained attention.

### 2.5 Sample size calculation

The variable "CPT Continuous Performance Test - Missing Number Component" was utilized for calculating the sample size. According to Zhou et al. (2020), the mean and standard deviation for the number of missing targets in COVID-19 patients were reported as 41.55 ± 2.90, while the control group exhibited a mean of 39.59 ± 2.31. Based on a statistical power of 95 % and a significance level (alpha) of 0.05, the calculated sample size for each group was determined to be 40 participants.

### 2.6 Statistical analysis

Descriptive statistics were employed to summarize the data, including means and standard deviations for continuous variables, and frequencies and percentages for categorical variables. Comparisons between groups for continuous variables were conducted using the independent *t*-test or its non-parametric equivalent, the Mann-Whitney *U* test. For comparisons involving more than two groups, one-way Analysis of Variance (ANOVA) or its non-parametric counterpart, the Kruskal-Wallis test, was applied. The Shapiro-Wilk test was utilized to assess the normality of continuous variables. Categorical variables were compared using the chi-square test or Fisher's exact test, as appropriate. All statistical analyses were performed using IBM SPSS Statistics Version 26.0, with a significance threshold set at p < 0.05.

## 3 Results

### 3.1 Study population characteristics

The characteristics of the study population are summarized in Table 1. The average ages of the COVID-19-infected and non-infected participants were 31.33 ± 5.94 and 33.31 ± 6.61 years, respectively, with no significant difference between the two groups (p = 0.164). There were also no significant differences between groups in terms of sex distribution (p = 0.496), marital status (p = 0.501), education level (p = 0.270), and occupational status (p = 0.805).

**Table 1:** The study population characteristics.

**Characteristics**		**Infected**	**Non-infected**	**P-value**
**Age**		31.33 ± 5.94	33.31 ± 6.61	0.164
**Sex**	Female	15 (37.5 %)	18 (55 %)	0.496
	Male	25 (62.5 %)	22 (45 %)	
**Education**	Diploma and less	5 (12.5 %)	11 (27.5 %)	0.270
	Associate	2 (5.0 %)	3 (7.5 %)	
	Bachelor	20 (50.0 %)	14 (35.0 %)	
	Master	7 (17.5 %)	3 (7.5 %)	
	Phd/doctorate	6 (15.0 %)	1 (2.5 %)	
**Marital status**	Single	23 (57.5 %)	20 (50 %)	0.501
	Married	17 (42.5 %)	20 (50 %)	
**Occupation**	Employed	28 (70.0 %)	29 (72.5 %)	0.805
	Unemployed	12 (30.0 %)	11 (27.5 %)	
**The number of infection**	1	8 (20.0 %)	-	-
	2	22 (55.0 %)	-	
	3 or more	10 (25.0 %)	-	
**The number of vaccine dose**	0	1 (2.5 %)	0 (0.0 %)	0.137
	1	1 (2.5 %)	0 (0.0 %)	
	2	20 (50.0 %)	29 (72.5 %)	
	3 or more	18 (45.0 %)	11 (27.5 %)	
**Anosmia**	No	11 (27.5 %)	-	-
	Yes	29 (72.5 %)	-	

Among the COVID-19-infected participants, the majority (n = 22, 55 %) reported experiencing the disease twice. Eight participants (20 %) had been infected once, while ten (25 %) reported three or more infections. Additionally, 29 participants (72.5 %) with COVID-19 experienced anosmia. The number of vaccine doses received by the two groups did not show a significant difference (p = 0.137).

### 3.2 Wisconsin test results

As presented in Table 2, the duration of the Wisconsin test was significantly longer for COVID-19-infected individuals (153.80 ± 47.39 s) compared to non-infected individuals (120.28 ± 35.39 s), with a statistically significant difference (p < 0.001). However, there were no significant differences between the two groups for other variables of the Wisconsin test.

**Table 2:** Comparing wisconsin test scores between COVID-19-infected and non-infected groups.

**Variables**	**Infected**		**Non-infected**		**P-value**
	**Mean ± SD**	**Median (Q1–Q3)**	**Mean ± SD**	**Median (Q1–Q3)**	
**Category number**	5.90 ± 0.38	6 (4–6)	5.98 ± 0.16	6 (5–6)	0.302
**Preservative errors**	0.95 ± 1.22	0.5 (0–4)	0.48 ± 0.75	0 (0–2)	0.077
**Total number of correct**	40.90 ± 3.71	40 (36–49)	39.68 ± 3.02	39 (36–47)	0.160
**Total number of errors**	8.80 ± 3.41	8 (5–18)	7.45 ± 2.39	7 (5–18)	0.079
**Total number of trials**	49.70 ± 6.26	49 (41–60)	47.13 ± 4.71	46 (41–60)	0.083
**Other errors**	7.85 ± 2.63	7.50 (5–15)	6.98 ± 1.97	7 (2–11)	0.156
**Duration of the test**	153.80 ± 47.39	143 (95–266)	120.28 ± 35.39	109.5 (90–268)	**< 0.001**
**Trials to complete the first category**	6.40 ± 1.46	1.46 (6–13)	6.00 ± 0.0	0 (0–1)	0.179
**Conceptual level responses**	6.00 ± 0.0	6 (6–6)	6.00 ± 0.0	6 (6–6)	> 0.990
**Failure to maintain set**	0.13 ± 0.34	0 (0–1)	0.08 ± 0.27	0 (0–1)	0.459

### 3.3 Stroop test findings

Table 3 displays the results of the Stroop test. The duration of the congruent test (p = 0.026) and the response time for congruent stimuli (p = 0.042) were significantly longer in COVID-19-infected participants compared to non-infected individuals. No significant differences were observed in other congruent and incongruent Stroop test variables (p > 0.05). The interference time for the Stroop test was significantly higher in the COVID-19-infected group (67.93 ± 29.68 s) compared to the non-infected group (52.50 ± 35.27 s) (p = 0.047).

**Table 3:** Comparing the stroop test scores between COVID-19-infected and non-infected groups.

**Variables**	**Infected**		**Non-infected**		**P-value**
	**Mean ± SD**	**Median (Q1–Q3)**	**Mean ± SD**	**Median (Q1–Q3)**	
**Congruent test duration**	44.7 ± 4.1	46 (35–51)	42.20 ± 5.30	43 (33–54)	**0.026**
**Number of congruent error**	0.43 ± 0.7	0 (0–3)	0.33 ± 0.57	0 (0–2)	0.491
**Number of congruent unanswered**	0.28 ± 0.5	0 (0–2)	0.08 ± 0.27	0 (0–1)	0.056
**Number of congruent correct**	47.30 ± 1.02	48 (43–48)	47.60 ± 0.59	48 (46–48)	0.194
**Congruent response time**	935.50 ± 81.52	954.5 (730–1046)	888.85 ± 109.56	904 (696–1144)	**0.042**
**Incongruent test duration**	47.10 ± 3.75	47.5 (39–54)	45.50 ± 5.33	46.5 (35–59)	0.146
**Number of incongruent error**	1.45 ± 2.24	1 (0–12)	0.70 ± 0.91	0 (0–3)	0.175
**Number of incongruent unanswered**	0.10 ± 0.30	0 (0–1)	0.10 ± 0.50	0 (0−3)	0.423
**Number of incongruent correct**	46.45 ± 2.39	47 (35–48)	47.20 ± 1.18	47.5 (42–48)	0.159
**Incongruent response time**	988 ± 76.72	990.5 (827–1127)	956.78 ± 107.64	974 (748–1207)	0.187
**Interference score**	0.85 ± 2.46	0 (− 5 to 11)	0.40 ± 1.30	0 (− 2 to 5)	0.483
**Interference time**	67.93 ± 29.68	66.50 (2–137)	52.50 ± 35.27	52.50 (− 19 to 152)	**0.047**

### 3.4 Digit span test results

As shown in Table 4, the length of the longest reread chain and the digit span test scores in the COVID-19-infected group were slightly lower than those of the non-infected group; however, these differences were not statistically significant (p > 0.05).

**Table 4:** Comparing digit span test scores between COVID-19-infected and non-infected groups.

**Variables**	**Infected**		**Non-infected**		**P-value**
	**Mean ± SD**	**Median (Q1–Q3)**	**Mean ± SD**	**Median (Q1–Q3)**	
Test score	8.93 ± 1.40	9 (6–12)	9.10 ± 1.37	9 (7–12)	0.578
The length of the longest chain read	7.13 ± 7.98	6 (5–56)	5.83 ± 0.72	6 (5–7)	0.942

**Table 5:** Comparison of continuous performance test scores in the first, second and third 50 stimuli between COVID-19-infected and non-infected groups.

**Variables**		**Infected**		**Non-infected**		**P-value**
		**Mean ±SD**	**Median (Q1–Q3)**	**Mean ± SD**	**Median (Q1–Q3)**	
First part	Error number	0.25 ± 0.45	0 (0–1)	0.33 ± 0.57	0 (0–2)	0.706
	Missing number	0.03 ± 0.16	0 (0–1)	0.08 ± 0.27	0 (0–1)	0.308
	Correct number	49.73 ± 0.45	50 (49–50)	49.60 ± 0.59	50 (48–50)	0.397
	Reaction time	454.03 ± 44.95	440.5 (392–607)	406.40 ± 13.19	404 (381–434)	**< 0.001**
Second part	Error number	0.20 ± 0.41	0 (0–1)	0.13 ± 0.34	0 (0–1)	0.366
	Missing number	0.08 ± 0.27	0 (0–1)	0.03 ± 0.16	0 (0–1)	0.308
	Correct number	49.37 ± 0.45	50 (49–50)	49.85 ± 0.36	50 (49–50)	0.174
	Reaction time	458.63 ± 48.05	452.5 (396–602)	410.23 ± 17.55	406.5 (368–454)	**< 0.001**
Third part	Error number	0.1 ± 0.3	0 (0–1)	0.3 ± 0.5	0 (0–1)	0.725
	Missing number	0.0 ± 0.0	0 (0–0)	0.1 ± 0.3	0 (0–1)	**0.041**
	Correct number	49.9 ± 0.3	50 (49–50)	49.6 ± 0.7	50 (48–50)	0.509
	Reaction time	442.9 ± 39.4	435.5 (391–549)	418.8 ± 22.1	412 (396–460)	**< 0.001**

### 3.5 Continuous performance test results

The reaction times in the Continuous Performance Test for the first, second, and third sets of 50 stimuli were significantly longer in the COVID-19-infected group compared to the non-infected group (p < 0.001). Additionally, a lower number of missing responses in the third set of 50 stimuli was observed in the COVID-19-infected group compared to the non-infected group (p = 0.041).

### 3.6 Gender-based analysis

Gender-based analysis within the COVID-19-infected group revealed that the length of the longest digit span chain in males (41.1 ± 38.9) was significantly greater than in females (18.1 ± 48.8). Furthermore, the digit span test score for males (34.7 ± 11.7) was significantly lower than that for females (61.0 ± 0.58) (p = 0.009). The number of missing responses in males (0 ± 0) was significantly lower than in females (33 ± 0.12) (p = 0.015). In contrast, the correct response rate in males (94.49 ± 0.25) was significantly higher than in females (70.49 ± 0.53) (p = 0.015).

### 3.7 Age-based analysis

Further analysis based on age indicated that participants older than 30 years exhibited significantly longer congruent test durations (43.73 ± 4.34 vs. 42.63 ± 5.39, p = 0.026), congruent response times (917.63 ± 88.44 vs. 895.53 ± 106.91, p = 0.042), and interference times (64.10 ± 31.64 vs. 53.77 ± 36.00, p = 0.047) compared to those younger than 30 years.

### 3.8 Olfactory function and cognition

Considering the potential relationship between olfactory function and cognitive performance, COVID-19-infected subjects with anosmia showed no significant differences in cognitive test outcomes compared to those without anosmia.

## 4 Discussion

The COVID-19 pandemic has led to a range of physical and mental health issues affecting individuals worldwide. Neglecting these issues may result in the psychological consequences of the COVID-19 epidemic persisting within the general population for an extended period, potentially evolving into a global medical crisis. The mid-term and long-term effects that survivors may experience are not yet well-defined and necessitate further longitudinal studies. This study investigated the cognitive performance of young adult patients at least four months post-recovery from mild COVID-19 compared to individuals who did not contract the virus. Our findings indicate impairments in processing speed and sustained attention among mild COVID-19 survivors up to four months after recovery. However, our subjects did not report functional impairment in their daily activities. Existing literature suggests a potential association between COVID-19 infection and an increased risk of cognitive decline following infection (Heneka et al., 2020). Our results support this hypothesis, revealing a consistent cognitive profile characterized by deficits in attention and executive function (EF), irrespective of initial symptom severity (ranging from asymptomatic to moderate/severe), particularly in older adults (Amalakanti et al., 2021, Miskowiak et al., 2021, Whiteside et al., 2021).

Our findings indicate that attention is one of the most significantly impaired cognitive functions following COVID-19. This observation is consistent with previous studies, the majority of which have reported attention deficits using various screening tests (Herrera et al., 2023, Bertuccelli et al., 2022). While this alignment reinforces the robustness of the observed attention impairments, the reliance on screening tools in many studies underscores the need for further validation through more comprehensive cognitive assessments. Additionally, in accordance with prior research, our study found impairments in processing speed, which have been correlated with disease severity (Cecchetti et al., 2022, Santoyo-Mora et al., 2022). However, the literature on chronic deficits in reaction time presents inconsistent results. Some studies indicate substantial impairments during the acute phase of COVID-19 (Becker et al., 2021), while others report weaker effects in severe cases or only among individuals with significant cognitive impairments (Santoyo-Mora et al., 2022). Furthermore, some online studies have found no significant differences in reaction times (Kelly et al., 2022). Similarly, another investigation revealed persistent deficits in perceptual processing speed among patients with post-COVID syndrome who reported cognitive complaints (Martin et al., 2024). These conflicting findings regarding psychomotor speed in post-COVID populations may arise from varying definitions of post-COVID conditions across different studies.

This research examined the potential relationship between anosmia (loss of smell) and cognitive function in individuals experiencing long COVID. Although the study did not establish a definitive connection, it recognizes prior research that suggests associations between olfactory ability and frontal lobe function, as well as between olfactory dysfunction and cognitive decline in neurodegenerative diseases (Doty, 2022, Vilarello et al., 2023). Furthermore, recent investigations have identified correlations between long COVID-related olfactory deficits and memory impairments, cognitive dysfunction, and persistent depressive symptoms (Llana et al., 2022, Simonini et al., 2024, Delgado-Alonso et al., 2022). The authors propose that, despite the absence of evidence for direct neural damage caused by COVID-19, the established link between olfaction and cognition in healthy individuals may account for the observed cognitive decline in some long COVID patients (Vilarello et al., 2023).

Since all participants in the present study were classified as having mild COVID-19, we were unable to examine the correlation between disease severity and the level of cognitive deficits. While numerous studies have investigated the relationship between COVID-19 and cognitive performance, the results remain inconclusive. Some investigations report no significant association between illness severity and cognitive impairment, while others indicate correlations between respiratory severity, lung function, and cognitive deficits, particularly affecting global cognition and executive function (Miskowiak et al., 2021, Alemanno et al., 2021, Woo et al., 2020). Our study adds to this evolving body of literature, as we observed better executive function in post-COVID-19 survivors than other investigations. Although this finding requires further investigation to confirm its validity, it may be supported by the mild severity of the disease, suggesting that lower severity of COVID-19 has a diminished impact on cognitive performance. Additionally, it is possible that younger age and higher levels of education serve as protective factors against cognitive impairment, as our patients had higher education levels and were in young ages. However, future prospective research specifically designed to assess cognitive performance and profiles across varying levels of disease severity, while considering the baseline characteristics of subjects, is essential to elucidate this relationship.

The strengths of the present study include its focus on cognitive function in patients with mild COVID-19 symptoms, in contrast to other studies that predominantly examined hospitalized patients with severe symptoms. Additionally, all participants were evaluated for major underlying mental and physical health conditions, which helps mitigate the influence of potential confounders. The Wisconsin Card Sorting Test and the Stroop Test were utilized to assess various cognitive domains, including conceptual reasoning, cognitive flexibility, selective attention, processing speed, and overall executive function.

However, several limitations should be acknowledged. Although the sample size was determined based on previous studies, a larger sample and long-term follow-up could enhance the validity of the results. Neuroimaging did not perform to rule out potential lesions in the brain and subjects included based on previous medical history indicating no significant neurological, internal, or psychiatric disorders. Furthermore, while there was no significant difference in the overall distribution of education levels between the two groups, individuals with academic and postgraduate degrees were more prevalent in the COVID-19-infected group, which could influence the study's findings. Therefore, investigating more homogeneous groups concerning age, education, and socioeconomic status—factors known to affect cognitive function—would yield more accurate and generalizable results.

## 5 Conclusion

The present study demonstrated that cognitive performance is impaired in individuals who have recovered from COVID-19. Various cognitive tests revealed decreased processing speed and sustained attention in patients with mild COVID-19 severity. These findings underscore the potential long-term cognitive effects of coronavirus infection in survivors.

It is recommended that healthcare systems incorporate routine cognitive assessments as part of follow-up care for individuals recovering from COVID-19. Such assessments could help identify and manage potential cognitive deficits that may impact mental health and overall quality of life.

## Authors' contributions

Design the research: F. A., A. E., and F. FH. Data collection: M.G., S. H., and M. E. Laboratory work: M. G. and A. HE Statistical analysis: S. H. Manuscript draft: F. A. and A. E. All authors helped edit and approve the final version of this manuscript for submission.

## CRediT authorship contribution statement

**Alireza Ebrahimi:** Writing – review & editing, Writing – original draft, Methodology, Formal analysis, Conceptualization. **Hajebikhanik Saeedeh:** Writing – review & editing, Writing – original draft, Methodology, Formal analysis. **Mojtaba ghalandarzadeh:** Writing – review & editing, Writing – original draft, Methodology, Formal analysis, Data curation. **Mahboubeh eslamzadeh:** Writing – review & editing, Writing – original draft, Methodology, Formal analysis. **Farhad Faridhosseini:** Writing – review & editing, Writing – original draft, Methodology, Investigation, Conceptualization. **Farzad Akbarzadeh:** Writing – review & editing, Writing – original draft, Methodology, Funding acquisition, Conceptualization.

## Ethics approval

The study was approved by the Ethical Committee of Mashhad University of Medical Sciences, Mashhad, Iran, with the Ethical approval number: IR.MUMS.MEDICAL.REC.1401.308.

## Ethics approval statement for work involving animals or human subjects

The study protocol was approved by the Ethics Committee of Mashhad University of Medical Sciences.

## Consent to participate

All participants signed a written informed consent approved in the Ethical Committee of Mashhad University of Medical Sciences.

## Patient consent statement for work with human subjects

All participants or their legal guardians provided written informed consent after being informed about the intervention procedures and potential side effects.

## Funding

This work was financially supported by 10.13039/501100004748Mashhad University of Medical Sciences under grant 4000762.

## Conflicts of Interest

The authors declare that they have no conflict of interest.

## Data Availability

The Data belongs to Mashhad University of Medical Sciences research council and would be available on request subjected to the current university regulations.
